# Supplementary material for: Collateral sensitivity networks reveal evolutionary instability and novel treatment strategies in ALK mutated non-small cell lung cancer
Source: Sci Rep. 2017 Apr 27;7:1232. doi: 10.1038/s41598-017-00791-8 (PMC5430816; doi:10.1038/s41598-017-00791-8)
Supplement: Supplementary file 1 — Supplementary Information [file 41598_2017_791_MOESM1_ESM.pdf]

Collateral sensitivity networks reveal evolutionary instability and novel treatment strategies in ALK mutated non-small cell lung cancer.

Andrew Dhawan+[1,2], Daniel Nichol+[3,4], Fumi Kinose[5], Mohamed E. Abazeed[1], Andriy Marusyk[6], Eric B. Haura[5], and Jacob G. Scott*[1]

[1]Department of Translational Hematology and Oncology Research, Cleveland Clinic
[2]Department of Oncology, University of Oxford
[3]Department of Computer Science, University of Oxford
[4]Department of Integrated Mathematical Oncology, H. Lee Moffitt Cancer Center and Research Institute
[5]Department of Thoracic Oncology, Experimental Therapeutics Program, H. Lee Moffitt Cancer Center and Research Institute
[6]Department of Cancer Imaging and Metabolism, H. Lee Moffitt Cancer Center and Research Institute
*scottj10@ccf.org
+these authors contributed equally to this work

Supplemental information

S1: Experimental Data and Code

All experimental data presented within this work and code for both data processing and figure generation is available at the GitHub repository at: https://github.com/andrewdhawan/alk-collateral-sensitivity (DOI: 10.5821/zenodo.61933).

S2: Variation of EC50 of treatment-naive cell line

To assess the baseline day-to-day variation in the measurement of the EC50 in the dose-response of the treatment-naive H3122 cell line to each drug, we present in Figure 1, the EC50 values and associated confidence intervals for each drug, taken against the treatment-naive cell line with three replicates, at different points in time (namely, the relaxation times). From this, we note that with the exception of alectinib, all other drugs show strong consistency in the measurement of the EC50, and alectinib moderate consistency. We note that for the case of alectinib, the degree of variation observed in the treatment-naive EC50s observed over the different time points along relaxation, is much less than the variation observed in the cases of significant cross-resistance when relaxing cell lines are treated with alectinib, underscoring that these significant interactions are not due to experimental noise.

S3: Alternative EC50 normalization

As we have shown in Supplementary Section S2, the baseline EC50 to which the EC50 of the relaxing cell lines is compared varies with time, and therefore may present an uneven baseline against which to normalize EC50 values. To address this variability, we consider an alternate normalization scheme, in which we consider an aggregate EC50 of treatment-naive cells treated with drug, by considering the overall average dose-response curve for all measurements of treatment-naive drug sensitivity for each drug over the course of the full experiment, and curve fit to obtain EC50 for this. We then consider the impact of this change in baseline EC50 on the relaxation heatmaps presented in Figure 2 in the main text. Recalculating these values and their significances, presented in Figure 2, we observe that the results are qualitatively quite similar to those presented in Figure 2 of the main text, giving further confidence in the treatment-naive EC50 measurements.

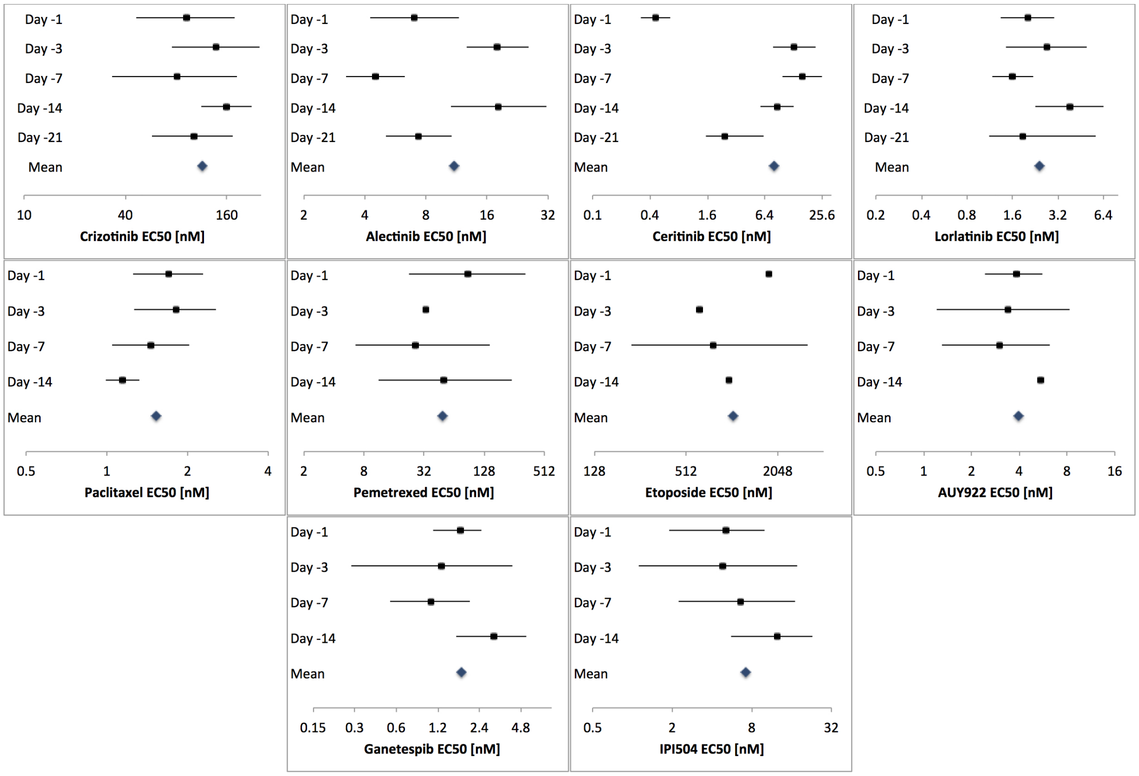

Figure 1. Forest plot of EC50 values and associated confidence intervals for the H3122 treatment-naive cell line, with drug sensitivities taken over the course of relaxation experiments. Confidence intervals are indicated as zero width in the case where it could not be calculated by the curve-fitting function.

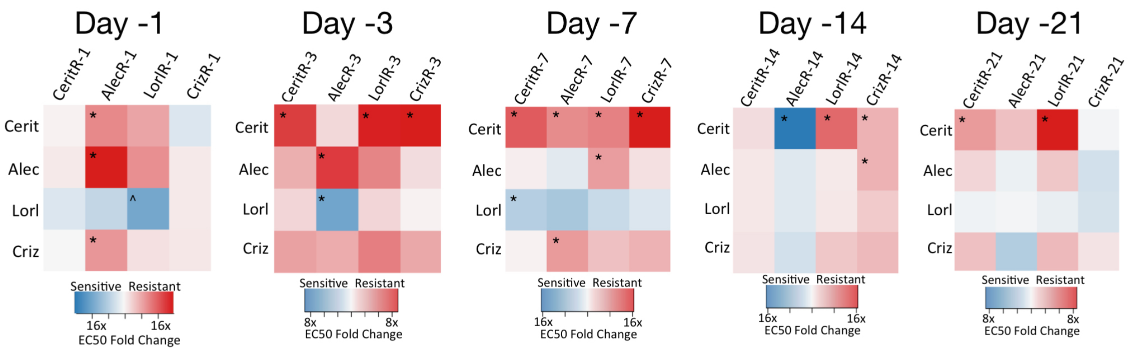

Figure 2. Collateral sensitivity matrices depicting fold change of EC50 for resistant cell lines, during the therapy holiday lasting for 1 day, 3 days, 7 days, 14 days, and 21 days, as treated with the panel of ALK TKIs (rows), as compared to the aggregate EC50. The asterisk (*) refers to a significant change in EC50 from treatment-naive, with non-overlapping 95% confidence intervals. Non-significant changes are those not demarcated by either symbol.
